# Quantum Epistemology and Falsification

**DOI:** 10.3390/e24040434

**Published:** 2022-03-22

**Authors:** Giacomo Mauro D’Ariano

**Affiliations:** 1 Dipartimento di Fisica dell’Università di Pavia, Via Bassi 6, 27100 Pavia, Italy; dariano@unipv.it; 2 Istituto Lombardo Accademia di Scienze e Lettere, 20121 Milano, Italy; 3 INFN, Gruppo IV, Sezione di Pavia, 27100 Pavia, Italy

**Keywords:** quantum theory axiomatization, Operational Probabilistic Theories (OPTs), randomness generation, falsifiability

## Abstract

The operational axiomatization of quantum theory in previous works can be regarded as a set of six epistemological rules for falsifying propositions of the theory. In particular, the Purification postulate—the only one that is not shared with classical theory—allows falsification of random-sequences generators, a task classically unfeasible.

## 1. Introduction

Our physical world is ruled by two theories: classical theory (CT) and quantum theory (QT). Compared to CT, QT still looks weird; however, this may be a symptom that we are still missing the hidden logic of the theory. Indeed, we should not forget that among the two theories, QT is the most powerful one, simply because CT is a restriction of QT. In fact, for given system dimension *d*, CT restricts QT’s states to the convex hull of a fixed maximal set of jointly perfectly discriminable pure states (the *d*-simplex), and, correspondingly, transformations are restricted to (sub)Markov linear maps. (For mathematical axiomatizations and main theorems of both theories, QT and CT, see Appendix A). We can thus regard the indeterminism inherent QT as the price to be payed for adding information-processing power.

Deriving QT from information-theoretical principles [1,2,3] reveals how the theory is more powerful than CT. Indeed, the two theories share five postulates, whereas the sixth QT postulate highlights the fundamental task that QT can achieve, whereas CT cannot: *purification*. On the other hand, the sixth CT postulate makes the theory a restriction of QT. Thus, purification synthesizes the additional power of QT compared to CT.

In the following, OPT will be the acronym for Operational Probabilistic Theory. (See Table A1 in Appendix A for acronyms, abbreviations, and symbols). OPTs have been introduced in Refs. [1,2,3], originally inspired by the works of L. Hardy [4] and C. Fuchs [5].

In the convex-OPT language, the five common postulates are:

P1 *Causality :* The probability of preparation is independent on the choice of observation.
P2 *Perfect discriminability:* Every state on the boundary of the convex set of states can be perfectly distinguished from some other state.
P3 *Local discriminability:* It is possible to discriminate any pair of states of composite systems using only local observations.
P4 *Compressibility:* For all states that are not completely mixed there exists an ideal compression scheme.
P5 *Atomicity of composition:* The composition of two atomic transformations is atomic.

The sixth postulate, different for each of the two theories, is:

P6_*Q*_ *Purification:* Every state has a purification. For a fixed purifying system, every two purifications of the same state are connected by a reversible transformation on the purifying system.
P6_*C*_ *Perfect joint discrimination:* For any system, all pure states can be perfectly discriminated jointly.

Notice that P6${}_{C}$ forces CT to restrict QT’s pure states to a maximal set of perfectly discriminable ones.

## 2. The Purification Principle

Let us recall the statement of the principle.

*For every system A and for every state ρ∈St(A), there exists a system B and a pure state Ψ∈PurSt(AB) such that:*


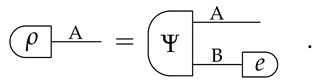

(1)

*If two pure states *Ψ* and $\Psi^{\prime }$ satisfy,*


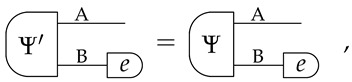


*then there exists a reversible transformation U, acting only on system B, such that:*


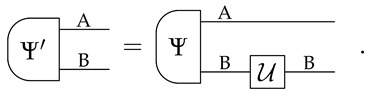

(2)



We call Ψ *a purification of* ρ, with B *purifying system*.

Informally, Equation (1) guarantees that we can always find a pure state of AB that is compatible with our limited knowledge of A alone. Furthermore, Equation (2) specifies that all the states of AB that are compatible with our knowledge of A are essentially the same, up to a reversible transformation on B. We call this property *uniqueness of purification*. (Note that the two purifications in Equation (2) have the same purifying system).

## 3. Epistemological Value of the Postulates

In quantum logic [6], one associates a “proposition” about the system A to an orthogonal projector $P_{S}$ on a subspace $S\subseteq H_{A}$ of the Hilbert space of A. With the map between $P_{S}$ and its support S being a bijection, one can equivalently associate “propositions” to Hilbert subspaces $S\subseteq H_{A}$. (Here, by “support” of an operator we mean the orthogonal complement of its kernel.) One can now enrich the notion of “proposition” by associating it to a quantum state ρ with support Supp ρ=S, the state ρ encoding a reacher information more than just its support S. We conclude that the notion of “state” constitutes a more detailed concept of “proposition” than that of the orthogonal projector. For this reason, in the present context, it is more appropriate to associate the word “proposition” to the notion of “quantum state” instead of the original definition as the orthogonal projector. In other words, a “proposition about A” will be a synonym of the “state of A”. Clearly, the same notion can be extended to CT, since CT is a restriction of QT.

According to the above identification we can now translate QT and CT into the language of “propositions”, and appreciate how, remarkably, all six postulates for QT and CT are of the epistemological nature, i.e., they all are assertions regarding falsifiability of the theory’s “propositions”.

### Epistemological Rules

E1 Causality is required for falsification of propositions by observations.
E2 Perfect discriminability guarantees the existence of falsifiable propositions derived from the theory.
E3 Local discriminability guarantees that falsifiability of joint propositions can be accomplished “locally”, namely using single system observations and classical communication.
E4 Compressibility provides the possibility of reducing the dimension of the system, supporting a falsifiable proposition.
E5 Atomicity of composition guarantees the existence of a class of transformations that do not affect falsifiability of propositions.
E6_*Q*_ Purification allows for falsifiable random generators.
E6_*C*_ Perfect discrimination allows any set of determinate propositions to be jointly falsifiable.

Any of the above principles constitutes an epistemological power of the corresponding principle. Statement E6${}_{Q}$, in particular, establishes the possibility of logically falsifying a quantum random generator, namely, there exists a falsification test that establishes if a given quantum random generator is different from a claimed one. Notice that such a falsification cannot be achieved classically for probabilities that are not deterministic, namely, p∉{0,1}, since no succession of outcomes can logically falsify a value of *p* different from 0 and 1. Remarkably, as we will see in this paper, thanks to the purification postulate, within QT we can falsify random generators with any probability distribution. Classically, one falsifies the generator for p=1 whenever the event does not occur, and for p=0 when it does. In such a case, the probability value itself is directly falsified. We stress that in the other cases, QT makes the random generator falsifiable—not the probability value.

Before proving the epistemological rules, we recall the theory of falsification tests introduced in Ref. [7].

## 4. The Falsification Test

We say that an event *F* is a *falsifier* of hypothesis Hyp if *F* cannot happen for Hyp=TRUE. We will call the binary test $\left\{F,F_{?}\right\}$ *a falsification test* for hypothesis Hyp, and denote by $F_{?}$ the *inconclusive event*. Notice that the occurrence of $F_{?}$ generally does not mean that Hyp=TRUE, but that Hyp has not been falsified.

Suppose now that one wants to falsify a proposition about the quantum state ρ∈St(A) of system A. In such case, any effective falsification test can be achieved as a binary *observation test* of the form:

(3) $$\left\{F,F_{?}\right\}\subset \mathrm{Eff}\left(A\right),\;F_{?}:=I_{A}-F,\;F>0,F_{?}\geq 0,$$

where by the symbol *F* ($F_{?}$) we denote both event and corresponding positive operator. The strict positivity of *F* is required for effectiveness of the test, F=0 corresponding to the *inconclusive test*, which outputs only the inconclusive outcome. On the other hand, $F_{?}=0$ corresponds to the logical a priori falsification.

Examples of inconclusive tests have been given in Ref. [7], to prove that hypotheses as “purity of an unknown state”, or “unitarity of an unknown transformation” cannot be falsified.

### Falsification of a Quantum State Support

Consider the proposition:

(4) $$\;\mathrm{Hyp}:\;\mathrm{Supp}\;\rho =:K⊊H_{A},\;\rho \in \mathrm{St}\left(A\right),\;\;\;\dim H_{A}\geq 2$$

Supp ρ denoting the support of ρ. Then, any operator of the form:

(5) $$0<F⩽I_{A},\;\mathrm{Supp}\;F\subseteq K^{⊥}$$

would have zero expectation for a state ρ satisfying Hyp in Equation (4), which means that occurrence of *F* would falsify Hyp, namely:

(6) Tr[ρF]>0 ⇒Hyp=FALSE.

Equation (5) provides the most general falsification test of Hyp in Equation (4), the choice $\mathrm{Supp}\;F=K^{⊥}$ corresponding to the most efficient test, namely the one maximizing falsification chance. Notice that the outcome corresponding to I−F does not correspond to a verification of Hyp, since it generally can occur for Supp (I−F)∩K≠∅.

## 5. Proofs of Epistemological Rules

In this section we prove the epistemological rules given in Section 3. We will denote by St(A) the convex set of states of system A and by ∂St(A) its boundary.

E1 For any proposition ρ that is falsifiable (i.e., $\mathrm{rank}\rho <d_{A}$), causality protects the falsification target state from being changed by the particular choice of observation.
E2 Consider two quantum states ρ,ν∈St(A). They are perfectly discriminable iff Supp ρ⊥Supp ν, which implies that $2\leq \mathrm{rank}\rho +\mathrm{rank}\nu \leq d_{A}$ with both ranks at least unit. It follows that $1\leq \mathrm{rank}\rho \leq d_{A}-\min\mathrm{rank}\nu =d_{A}-1$ and the same for rankν, hence both ρ,ν∈∂St(A). We conclude that the two states are falsifiable, with falsifiers kerρ⊆Supp ν and kerν⊆Supp ρ, respectively.
E3 Here, we will use the *double-ket* notation [8] (for a thorough treatment see [3]). Shortly, once it is chosen the orthonormal factorized canonical basis {|i〉⊗|j〉} for H⊗H, the one-to-one correspondence between vectors in H⊗H and operators on H holds
  $$|\Psi \rangle \rangle :=\sum_{ij}\Psi_{ij}|i\rangle ⊗|j\rangle \;⟷\;\Psi =\sum_{ij}\Psi_{ij}\left|i\rangle \langle j\right|\in \mathrm{HS}\left(C^{d}\right),$$
  where $\mathrm{HS}(C^{d})$ denotes the Hilbert–Schmidt operators in dimensions *d*. One can then veryify the following identity:
  $$\left(A⊗B\right)|C\rangle \rangle =|ACB^{⊺}\rangle \rangle ,$$
  with $B^{⊺}$ denoting the *transposed operator* of *B*, the operator that has the transposed matrix w.r.t. the canonical basis. Notice that e.g., ${\left(|\phi \rangle \langle \psi |\right)}^{⊺}=|\psi^{*}\rangle \langle \phi^{*}|$ where $|\psi^{*}\rangle$ is the vector |ψ〉 with complex-conjugated coefficients w.r.t. the canonical basis.
  Consider now the pure entangled state corresponding to state-vector $|A\rangle \rangle \in H_{A}⊗H_{B}$. The following sequence of identities holds:
  (7) $$\left(\langle a\right|⊗\langle b|)|A\rangle \rangle ={(\langle a|⊗I)(I⊗\langle b|A^{T})\sum_{n}|a_{n}\rangle ⊗|a_{n}\rangle }^{*}=\langle b|A^{T}{(|a\rangle }^{*})={\langle b|(A^{†}|a\rangle )}^{*}$$
  where $S:=\{|a_{n}\rangle {\}}_{n=1}^{d_{A}}$ is an orthonormal basis for $H_{A}$, with |a〉∈S. Equation (7) shows that choosing |b〉 orthogonal to $(A^{†}|a\rangle {)}^{*}$ one has a local falsifier of the entangled state |A〉〉〈〈A| given by:
  (8) $$P_{A}⊗P_{B}=\left|a\rangle \langle a\right|⊗|b\rangle \langle b|,$$
  One can see that the generalization to mixtures *R* is straightforward, upon writing the state *R* in the canonical form:
  (9) $$R=\sum_{j=1}^{d_{A}}|A_{j}\rangle \rangle \langle \langle A_{j}|,\;\mathrm{Tr}\left(A_{i}^{†}A_{j}\right)=\delta_{ij}p_{j},\;\sum_{j=1}^{d_{A}}p_{j}=1.$$
E4 Any falsifiable state ρ has dimkerρ≥1, hence it can be isometrically mapped to a state of a system B with $d_{B}\leq d_{A}-1$.
E5 A transformation A∈Trn(A→B) is called *atomic* if it has only one Krauss term, namely it can be written as $A\rho =A\rho A^{†}$, with $A\in B(H_{A})$ and ||A||≤1. This implies that rank(Aρ)≤rankρ, namely the falsification space has a dimension that is not decreased; hence, the output state Aρ can be falsified. This is not necessarily true for A *non atomic*, namely with more than one Krauss term, i.e., $A\rho =A_{1}\rho A_{1}^{†}+A_{2}\rho A_{2}^{†}+\ldots$
E6_*Q*_ See Section 6.
E6_*C*_ It trivially holds for CT.

## 6. Falsifiable Setup of a Random Generator

A falsifiable setup for a quantum binary random generator can use any quantum system A, e.g., a qubit, in a pure state ρ=|ψ〉〈ψ| along with an orthogonal observation test $\Omega ={\left\{\omega_{i}\right\}}_{i=0,1}$ with $\omega_{i}=\left|i\rangle \langle i\right|$, $\langle i|j\rangle =\delta_{ij}$ (namely a “customary discrete” observable). The following setup,

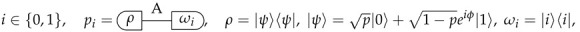
(10)
is a binary random generator with probability $p_{0}=p$.

Notice that the advantage of this choice of setup (compared to, e.g., using a mixed state and/or a non orthogonal observation test) is that it is falsifiable. In fact, the preparation of the state |ψ〉〈ψ| can be falsified efficiently by running the falsification test of the state support, using falsifier F=I−|ψ〉〈ψ|. On the other hand, the observation test Ω can be taken as just the observable providing the physical meaning of the orthonormal basis chosen for the qubit A (e.g., spin-up and spin-down), which is required to physically define the state preparation.

The above setup can be trivially generalized to a *N*-ary random generator (N>2) with probability distribution ${\left\{p_{n}\right\}}_{n\in Z_{N}}$ by using a system A with $\dim(H_{A})=N$, and a pure state with vector with more than two nonvanishing probability amplitudes. Here, F=I−|ψ〉〈ψ| still provides the most efficient falsifier. Notice that for $\dim(H_{A})>2$ it is also possible to falsify mixed states with rank strictly smaller than $\dim(H_{A})$. Notice that the probability of falsification of a mixed state ρ≠|ψ〉〈ψ| is given by p=1−〈ψ|ρ|ψ〉, and vanishes linearly with the overlap between the declared state |ψ〉〈ψ| and the true state ρ.

## 7. Conclusions

CT and QT are more than theories about the world: they constitute extensions of logic. Famously, von Neumann attempted to prove QT to be a kind of logic: we now know that it is an extension of it, instead. We have seen that QT can be regarded as a chapter of epistemology, being a set of rules for accessibility of falsifications. Thus, more than answering the question “what is reality”, QT provides rules for “how we can explore reality”. One can then add axioms to those of QT to get more refined theories, such as Free Quantum Field Theory. The latter can indeed be obtained upon considering a denumerable set of QT systems, and adding the axioms of locality, homogeneity, and isotropy of interactions (see, e.g., the review [9]).

## Data Availability

Not applicable.

## Appendix A. Quantum and Classical Theories: Axiomatization and Main Theorems

Minimal mathematical axiomatizations of QT and CT as OPTs are provided in Table A2 and Table A4. For an OPT, we need to provide the mathematical description of systems, their composition, and transformations from one system to another (the rules of compositions of transformations and their respective systems are provided by the OPT framework). The reader who is not familiar with the OPT framework can simply use the intuitive construction of quantum circuits. In Table A3 and Table A5 we report the main theorems following from the axioms. For the interested reader, the motivations for adopting the mathematical axiomatizations in Table A2 and Table A4 are discussed in ref. [7].

**Table A1:** Notation, special-cases corollaries, and common abbreviations.

$B^{+}\left(H\right)$	bounded positive operators over H
${\mathrm{CP}}_{\leq }$	trace-non increasing completely positive map
${\mathrm{CP}}_{=}$	trace-preserving completely positive map
H	Hilbert space over C
Cone(S)	conic hull of S
${\mathrm{Cone}}_{\leq 1}\left(S\right)$	convex hull of {S∪0}
Conv(S)	convex hull of S
Eff(A)	set of effects of system A
${\mathrm{Eff}}_{1}\left(A\right)$	set of deterministic effects of system A
${\mathrm{Mrkv}}_{\leq }$	normalization-non-incressing right-stochastic Markov matrices
${\mathrm{Mrkv}}_{1}$	normalization-preserving right-stochastic Markov matrices
Prm(n)	n×n permutation matrices
${\left(R^{n}\right)}_{\leq 1}^{+}$	$\left\{x\in R^{n_{A}}|x\geq 0,x\leq 1\right\}$: simplex $S^{n_{A}+1}$
${\left(R^{n}\right)}_{1}^{+}$	$\left\{x\in R^{n_{A}}|x\geq 0,||x|{|}_{1}=1\right\}$: simplex $S^{n_{A}}$
St(A)	set of states of system A
${\mathrm{St}}_{1}\left(A\right)$	set of deterministic states of system A
T(H)	trace-class operators over H
$T^{+}\left(H\right)$	trace-class positive operators over H
$T_{\leq 1}^{+}\left(H\right)$	positive sub-unit-trace operators over H
$T_{=1}^{+}\left(H\right)$	positive unit-trace operators over H
Trn(A→B)	set of transformations from system A to system B
${\mathrm{Trn}}_{1}\left(A\to B\right)$	set of deterministic transformations from system A to system B
U(H)	unitary group over H
	**Special cases corollaries**
	T(C)=C, $T^{+}\left(C\right)=R^{+}$, $T_{\leq 1}^{+}\left(C\right)=\left[0,1\right]$, $T_{=1}^{+}\left(C\right)=\left\{1\right\}$
	$\mathrm{CP}\left(T\left(H\right)\to T\left(C\right)\right)=P\left(T\left(H\right)\to T\left(C\right)\right)=\left\{\mathrm{Tr}\left[\cdot E\right],\;E\in B^{+}\left(H\right)\right\}$
	$\mathrm{CP}\left(T\left(C\right)\to T\left(H\right)\right)=P\left(T\left(C\right)\to T\left(H\right)\right)=T^{+}\left(H\right)$
	${\mathrm{CP}}_{\leq }\left(T\left(C\right)\to T\left(H\right)\right)\equiv T_{\leq 1}^{+}\left(H\right)$
	${\mathrm{CP}}_{\leq }\left(T\left(H\right)\to T\left(C\right)\right)\equiv \left\{\epsilon \left(\cdot \right)=\mathrm{Tr}\left[\cdot E\right],\;0\leq E\leq I\right\}$
	${\mathrm{Mrkv}}_{\leq }\left(n,1\right)={\left(R^{n}\right)}_{\leq 1}^{+}$
	${\mathrm{Mrkv}}_{1}\left(n,1\right)={\left(R^{n}\right)}_{=1}^{+}$

**Table A2:** Mathematical axiomatization of Quantum Theory. As given in the table, in Quantum Theory to each system A, we associate a Hilbert space over the complex field $H_{A}$. To the composition of systems A and B, we associate the tensor product of Hilbert spaces $H_{AB}=H_{A}⊗H_{B}$. Transformations from system A to B are described by trace-nonincreasing completely positive (CP) maps from traceclass operators on $H_{A}$ to traceclass operators on $H_{B}$. Special cases of transformations are those with input trivial system I corresponding to states, whose trace is the preparation probability, the latter providing an efficient Born rule from which one can derive all joint probabilities of any combination of transformations. Everything else is simply special-case corollaries and one realization theorem: these are reported in Table A3.

Quantum Theory		
system	A	$H_{A}$
system composition	AB	$H_{AB}=H_{A}⊗H_{B}$
transformation	T∈Trn(A→B)	$T\in {\mathrm{CP}}_{\leq }\left(T\left(H_{A}\right)\to T\left(H_{B}\right)\right)$
Born rule	p(T)=TrT	T∈Trn(I→A)

**Table A3:** Corollaries and a theorem of Quantum Theory, starting from Table A2 axiomatization. The first corollary states that the trivial system I, in order to satisfy the composition rule IA=AI=A, must be associated with the one-dimensional Hilbert space $H_{I}=C$, since it is the only Hilbert space that trivializes the Hilbert space tensor product. The second corollary states that the reversible transformations are the unitary ones. The third corollary states that the deterministic transformations are the trace-preserving ones. Then the fourth and fifth corollaries give the composition of transformations in terms of compositions of maps. We then have four corollaries about states: (1) states are transformations starting from the trivial system and, as such, are positive operators on the system Hilbert space, having trace bounded by one; (2) the deterministic states correspond to unit-trace positive operator; (3) the states of the trivial system are just probabilities; (4) the only deterministic state of the trivial system is number 1. We then have two corollaries for effects, as special cases of transformation toward the trivial system: (1) the effect is represented by the partial trace over the system Hilbert space of the multiplication with an operator that is positive and bounded by the identity; (2) the only deterministic effect is the partial trace over the system Hilbert space. Finally, we have the realization theorem for transformations in terms of unitary interaction $U=U\cdot U^{†}$ with an environment F and a projective effect-test $\left\{P_{i}\right\}$ over environment E, with $P_{i}=P_{i}\cdot P_{i}$, $\left\{P_{i}\right\}$ being a complete set of orthogonal projectors.

Quantum Theorems		
trivial system	I	$H_{I}=C$
reversible transf.	$U=U\cdot U^{†}$	$U\in U(H_{A})$
determ. transformation	$T\in {\mathrm{Trn}}_{1}\left(A\to B\right)$	$T\in {\mathrm{CP}}_{\leq 1}\left(T\left(H_{A}\right)\to T\left(H_{B}\right)\right)$
parallel composition	$T_{1}\in \mathrm{Trn}\left(A\to B\right)$, $T_{2}\in \mathrm{Trn}\left(C\to D\right)$	$T_{1}⊗T_{2}$
sequential composition	$T_{1}\in \mathrm{Trn}\left(A\to B\right)$, $T_{2}\in \mathrm{Trn}\left(B\to C\right)$	$T_{2}T_{1}$
states	ρ∈St(A)≡Trn(I→A)	$\rho \in T_{\leq 1}^{+}\left(H_{A}\right)$
	$\rho \in {\mathrm{St}}_{1}\left(A\right)\equiv {\mathrm{Trn}}_{1}\left(I\to A\right)$	$\rho \in T_{=1}^{+}\left(H_{A}\right)$
	ρ∈St(I)≡Trn(I→I)	ρ∈[0,1]
	$\rho \in {\mathrm{St}}_{1}\left(I\right)\equiv \mathrm{Trn}\left(I\to I\right)$	ρ=1
effects	ϵ∈Eff(A)≡Trn(A→I)	$\epsilon \left(\cdot \right)={\mathrm{Tr}}_{A}\left[\cdot E\right],\;0\leq E\leq I_{A}$
	$\epsilon \in {\mathrm{Eff}}_{1}\left(A\right)\equiv {\mathrm{Trn}}_{1}\left(A\to I\right)$	$\epsilon ={\mathrm{Tr}}_{A}$
Transformations as unitary interaction + von Neumann-Luders		$T_{i}\rho ={\mathrm{Tr}}_{E}\left[U\left(\rho ⊗\sigma \right)U^{†}\left(I_{B}⊗P_{i}\right)\right]$
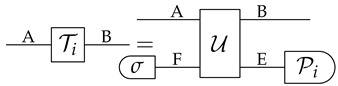

**Table A4:** Mathematical axiomatization of Classical Theory. To each system A, we associate a real Euclidean space $R^{n_{A}}$. To the composition of systems A and B, we associate the tensor product spaces $R^{n_{A}}⊗R^{n_{B}}$. Transformations from system A to system B are described by substochastic Markov matrices from the input space to the output space. All others are simple special-case corollaries: these are reported in Table A5.

Classical Theory		
system	A	$R^{n_{A}}$
system composition	AB	$R^{n_{AB}}=R^{n_{A}}⊗R^{n_{B}}$
transformation	T∈Trn(A→B)	$T\in {\mathrm{Mrkv}}_{\leq }\left(R^{n_{A}},R^{n_{B}}\right)$

**Table A5:** Main theorems of Classical Theory, starting from axioms in Table A4. The first corollary states that the trivial system I in order to satisfy the composition rule IA=AI=A must be associated to the one-dimensional space R, since it is the only real linear space that trivializes the tensor product. The second corollary states that the reversible transformations are the permutation matrices. The third states that transformations are substochastic Markov matrices. The fourth states that the deterministic transformations are stochastic Markov matrices. Then the fifth and sixth corollaries give the composition of transformations in terms of composition of matrices. We then have four corollaries about states: (1) states are transformations starting from the trivial system and, as such, are sub-normalized probability vectors (vectors in the positive octant with sum of elements bounded by one); (2) the deterministic states correspond to normalized probability vectors; (3) the case of trivial output-system correspond to just probabilities; (4) the only trivial output-system deterministic state is the number 1. We then have two corollaries for effects, as special cases of the transformation toward the trivial system: (1) the effect is represented by scalar product with a vector with components in the unit interval; (2) the only deterministic effect is the scalar product with the vector with all unit components.

Classical Theorems		
trivial system	I	R
reversible transformations	P	$P\in \mathrm{Prm}(n_{A})$
transformation	$T\in {\mathrm{Trn}}_{⩽}\left(A\to B\right)$	$T\in {\mathrm{Mrkv}}_{⩽}\left(R^{n_{A}},R^{n_{B}}\right)$
determ. transformation	$T\in {\mathrm{Trn}}_{1}\left(A\to B\right)$	$T\in {\mathrm{Mrkv}}_{1}\left(R^{n_{A}},R^{n_{B}}\right)$
parallel composition	$T_{1}\in \mathrm{Trn}\left(A\to B\right)$, $T_{2}\in \mathrm{Trn}\left(C\to D\right)$	$T_{1}⊗T_{2}$
sequential composition	$T_{1}\in \mathrm{Trn}\left(A\to B\right)$, $T_{2}\in \mathrm{Trn}\left(B\to C\right)$	$T_{2}T_{1}$
states	x∈St(A)≡Trn(I→A)	$x\in {\left(R^{n_{A}}\right)}_{\leq 1}^{+}$
	$x\in {\mathrm{St}}_{1}\left(A\right)\equiv {\mathrm{Trn}}_{1}\left(I\to A\right)$	$x\in {\left(R^{n_{A}}\right)}_{=1}^{+}$
	p∈St(I)≡Trn(I→I)	p∈[0,1]
	$p\in {\mathrm{St}}_{1}\left(I\right)\equiv \mathrm{Trn}\left(I\to I\right)$	p=1
effects	ϵ∈Eff(A)≡Trn(A→I)	ϵ(·)=·x, 0≤x≤1
	$\epsilon \in {\mathrm{Eff}}_{1}\left(A\right)\equiv {\mathrm{Trn}}_{1}\left(A\to I\right)$	ϵ=·1

## References

[1] Chiribella G., D’Ariano G.M., Perinotti P. (2010). Probabilistic theories with purification. Phys. Rev. A.

[2] Chiribella G., D’Ariano G.M., Perinotti P. (2011). Informational derivation of quantum theory. Phys. Rev. A.

[3] D’Ariano G.M., Chiribella G., Perinotti P. (2017). Quantum Theory from First Principles.

[4] Hardy L. (2001). Quantum Theory From Five Reasonable Axioms. arXiv.

[5] Fuchs C.A. (2002). Quantum Mechanics as Quantum Information (and only a little more). arXiv.

[6] Birkhoff G., Von Neumann J. (1936). The logic of quantum mechanics. Ann. Math..

[7] D’Ariano G.M. (2020). No purification ontology, no quantum paradoxes. Found. Phys..

[8] D’Ariano G.M., Lo Presti P., Sacchi M. (2002). A quantum measurement of the spin direction. Phys. Lett. A.

[9] D’Ariano G.M. (2017). Physics without physics: The power of information-theoretical principles. Int. J. Theor. Phys..

